# A bibliometric visualization of resistance to lung cancer immunotherapy: a decade of research progress (2014–2024)

**DOI:** 10.3389/fonc.2025.1656967

**Published:** 2025-11-10

**Authors:** Zhuo Yang, Lanlan Yang, Yuli Wang, Xiangyu Ren, Yajing Cui, Yan Li, Jianchun Wu

**Affiliations:** Clinical Medical Center of Oncology, Shanghai Municipal Hospital of Traditional Chinese Medicine, Shanghai University of Traditional Chinese Medicine, Shanghai, China

**Keywords:** bibliometric, lung cancer, immunotherapy resistance, mechanisms, tumor microenvironment

## Abstract

**Background:**

Lung cancer remains the leading cause of cancer-related deaths globally and represents the most common malignant tumor. While immunotherapy has significantly improved patient survival in recent years, the development of resistance limits its clinical efficacy. Currently, a systematic and comprehensive bibliometric analysis of drug resistance in immunotherapy for lung cancer is lacking. This study aims to address this gap by employing bibliometric methods to illuminate the knowledge structure and to identify key research hotspots in this critical area.

**Methods:**

We retrieved publications concerning lung cancer immunotherapy drug resistance from the Web of Science Core Collection and PubMed databases, covering January 1, 2014, to December 31, 2024. NoteExpress was used for data integration, duplicate detection, and screening. Subsequently, we quantitatively and visually analyzed the characteristics of the selected literature, with an emphasis on country, institution, and keywords. This analysis was performed utilizing VOSviewer, CiteSpace, and the “bibliometrix” package in R.

**Result:**

The annual publication output showed a marked upward trend, peaking in 2024. China produced the most publications, while the USA demonstrated higher citation impact. Analysis of keywords revealed a clear thematic evolution: from initial focus on clinical trials (e.g. Open-label) and specific drugs (e.g. Nivolumab), to immune checkpoints (e.g.PD-1/PD-L1), and more recently to underlying molecular mechanisms like the tumor microenvironment, autophagy, and ferroptosis.

**Conclusions:**

This study offers a thorough overview of the most important research topics and emerging trends related to drug resistance and lung cancer immunotherapy. By integrating current knowledge, it enables researchers to swiftly identify pivotal research directions, thereby promoting in-depth development and innovation within the field and supporting the progression of clinical practice. For clinicians, this bibliometric insight provides a more scientific and precise basis for formulating treatment strategies, ultimately assisting lung cancer patients in deriving benefits from immunotherapy.

## 1 Introduction

According to the most recent data from the International Agency for Research on Cancer, lung cancer has emerged as the leading global threat to cancer-related morbidity and mortality. In 2022, nearly 2.5 million new cases were diagnosed worldwide, representing 12.4% of all cancer cases, and it caused approximately 1.8 million deaths, accounting for 18.7% of total cancer fatalities (1). Despite advances in diagnostics and treatment options, managing lung cancer remains highly challenging. Radical surgery is effective primarily for early-stage patients without metastasis; however, about 48% of patients already present with distant metastasis at diagnosis, resulting in a dismal 5-year relative survival rate of only 8% (2). This stark reality underscores the urgent necessity of developing more effective therapeutic strategies to enhance patient outcomes.

According to the World Health Organization’s 5th edition Thoracic Tumors Classification, the classification of lung cancer distinguishes between two primary groups: small cell lung cancer (SCLC) and non-small cell lung cancer (NSCLC). NSCLC includes multiple subtypes (3) including lung adenocarcinoma, lung squamous cell carcinoma, and large cell lung carcinoma lung cancer. NSCLC accounts for over 85% of lung cancer cases and is the most common type of lung cancer, while SCLC accounts for approximately 15% of lung cancer cases and is highly invasive and prone to early metastasis, rendering it particularly difficult to treat clinically (4). Prior to the advent of immunotherapy, chemotherapy constituted the principal therapeutic approach for managing lung cancer, with the objective of controlling tumor proliferation and preventing recurrence or metastasis (5).Nonetheless, chemotherapy is frequently linked to significant toxicities and adverse side effects, which can adversely affect patient adherence and quality of life. In contrast, immunotherapy serves as a promising alternative by stimulating immune response to target cancer cells with precision, thereby mitigating some of the toxicity issues associated with conventional chemotherapy (6).

Cancer immunotherapy offers a promising new avenue for lung cancer treatment by harnessing the body’s immune system to target and eliminate tumor cells. This approach activates T cell-mediated responses against tumor-specific antigens (TSA) and tumor-associated antigens (TAA) (5). Immune checkpoint inhibitors (ICIs), which target molecules such as programmed death receptor 1 (PD-1), programmed death ligand 1 (PD-L1), and cytotoxic T-lymphocyte-associated antigen 4 (CTLA-4), have shown significant benefits in improving overall survival (OS) for patients with advanced NSCLC. For some patients, the 5-year survival rate has increased from approximately 5% with conventional chemotherapy to as high as 21.9% when ICIs are incorporated into treatment regimens (7). In extensive-stage SCLC, phase III clinical trials such as the IMpower133 study have demonstrated that combining ICIs with chemotherapy extends median OS from 10.3 months to 12.3 months and reduces the risk of death by 30%. This represents a breakthrough advancement in the immunotherapy treatment landscape for SCLC, offering new hope for improved patient outcomes (8).

With the expanding application of immunotherapy in clinical practice, the challenge of immunotherapeutic drug resistance has gained increasing prominence. Research indicates that over 60% of patients develop acquired resistance after initial treatment with PD-L1 inhibitors (9). Despite notable advances in immunotherapy, resistance mechanisms significantly affect the prognosis of lung cancer patients, heightening their risk of disease progression or recurrence. Consequently, a comprehensive understanding of the underlying mechanisms driving immunotherapy resistance in lung cancer is imperative for the development of effective treatment and improved patient outcomes.

While previous bibliometric studies have mapped the broader landscape of cancer immunotherapy or lung cancer research, a focused, decade-long analysis specifically targeting the evolving domain of drug resistance to immunotherapy in lung cancer is currently lacking. Existing reviews often concentrate on biological mechanisms or clinical management, leaving a gap in our quantitative understanding of the global research architecture, collaborative networks, and intellectual turning points within this subfield. This study aims to fill this gap by conducting the first comprehensive bibliometric analysis dedicated to lung cancer immunotherapy resistance from 2014 to 2024. We seek not only to delineate the quantitative contributions of countries, institutions, and journals but also to decode the thematic evolution and emergent frontiers that have defined the past decade. By integrating quantitative metrics with qualitative interpretation, this review provides a unique lens through which to view the past, present, and future of overcoming one of the most pressing challenges in thoracic oncology.

The aim of this study is to (1) reveal the development of the field and the scientific contributions of national institutions; (2) analyze the key research forces (countries, institutions, authors) and high-impact results; (3) clarify the progress of the knowledge structure of the core topics such as resistance mechanisms, biomarkers, and reversal strategies; (4) provide directional suggestions for future breakthroughs in immunoresistance, and provide theoretical support for the development of clinical translational and precision therapeutic strategies.

## 2 Materials and methods

### 2.1 Data retrieval

A systematic search of literature related to lung cancer immunotherapeutic resistance was conducted between January 1,2014 and December 31, 2024, in the Web of Science Core Collection and Pubmed. The search strategy utilized the following terms: TS=(“lung cancer”OR”lung carcinoma”) AND TS=(“immunotherapy resistance*”OR”drug resistance inimmunotherapy”).

The search was limited to articles and reviews in English. Extracted records and references from the search results have been stored in plain text format to facilitate future analysis. NoteExpress was used for duplicate checking and data filtering. The exclusion criteria are as follows: First, documents that are not related to the topic (such as those that only mention immunotherapy but not lung cancer, or those that only study lung cancer treatment but have nothing to do with immunotherapy); second, publications of other types except for research articles and reviews. The literature retrieval and screening process was independently conducted by two authors. Any differences shall be resolved through consultation with the third author. A total of 2,532 papers were included in the final dataset, comprising 1,369 articles and 1,163 reviews.

**Figure 1:**
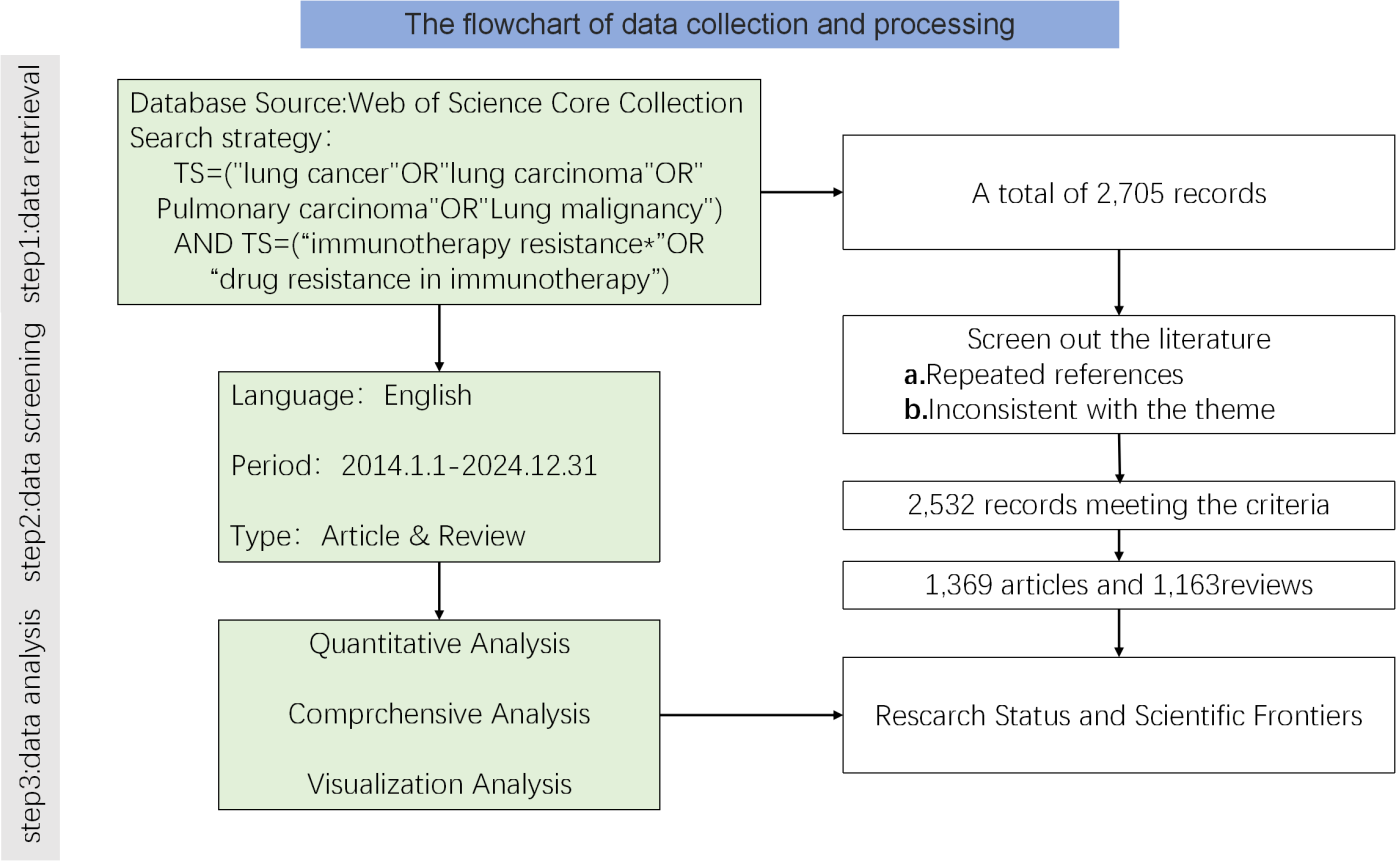
Flowchart of the literature search and selection process for studies on drug resistance to immunotherapy in lung cancer.

A flow diagram of the literature search and selection process **Figure 1** was created following the PRISMA guidelines.

### 2.2 Variables and analysis

For the analyzed publications, various attributes were extracted and examined, including authorship, country of origin, institutional affiliation, journal titles, and keywords. To facilitate data visualization and analysis, multiple bibliometric tools were employed: the Bibliometrix package in R software (version 4.4.0), VOSviewer (version 1.6.17), and CiteSpace (version 6.3.1). Utilizing these tools, a comprehensive visualization of the data was created to reveal patterns and relationships within the research landscape. Specifically, CiteSpace was used to conduct an in-depth analysis of keyword trends over time, allowing us to identify emerging frontiers and pivotal themes in the field of immunotherapy resistance in lung cancer. Data from the 2023 Journal Citation Reports (JCR), including Impact Factor (IF) values, was incorporated into the analysis to assess the scientific influence and prestige of the journals involved, providing valuable context for the quality and impact of the published research.

### 2.3 Some parameter thresholds for analysis

#### 2.3.1 Co-authorship/collaboration analysis (countries/institutions)

A minimum number of 20 documents per country/institution was set to identify significant entities. Keyword Analysis: A minimum occurrence threshold of 10 was applied to filter out insignificant terms and focus on the most representative research hotspots. Co-citation Analysis (References): A minimum citation count of 10 was set for a reference to be included in the network, ensuring the analysis captures the core knowledge base of the field.

## 3 Results

### 3.1 Annual growth trend of publications

**Figure 2:**
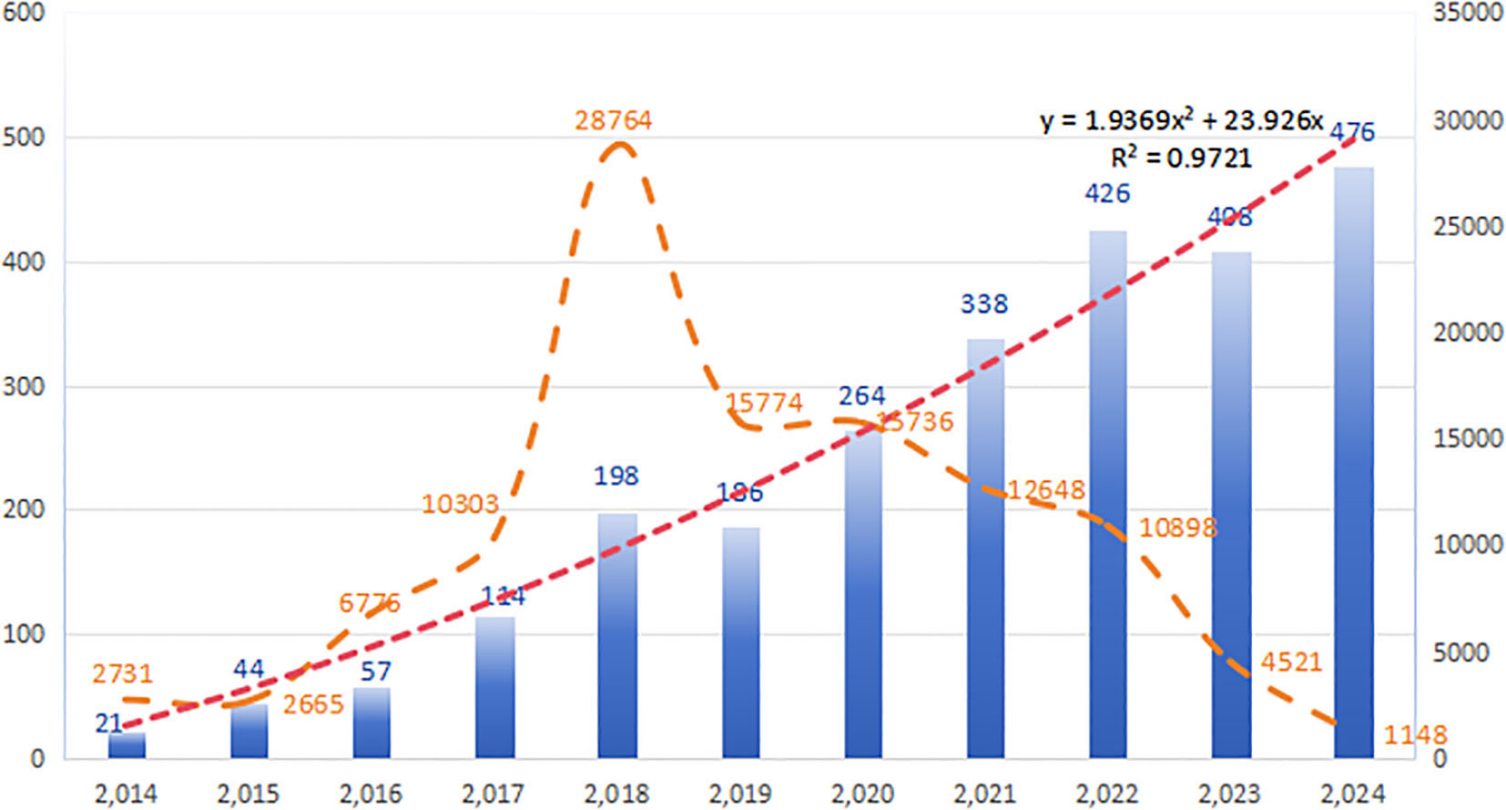
Publication and annual citation trends.

Between January 1, 2014, and December 31, 2024, a total of 2,532 publications on resistance of immunotherapy in lung cancer were identified, comprising 1,163 reviews (45.94%) and 1,369 research articles (54.06%). The literature search and screening process is outlined in **Figure 1**. **Figure 2** depicts the annual publication rate from 2014 to 2024, alongside the trends in corresponding citations. A peak in citations is observed in 2018, signifying a potential turning point or significant advancement in the field. This observation highlights the increasing importance of immunotherapy resistance as a key area of investigation in oncology. The number of publications in this field has consistently increased year-over-year, reaching a peak of 476 in 2024. Based on a linear fitting of this trend, the number of publications in this field is projected to exceed 550 by 2025.

### 3.2 Analysis of countries

**Figure 3:**
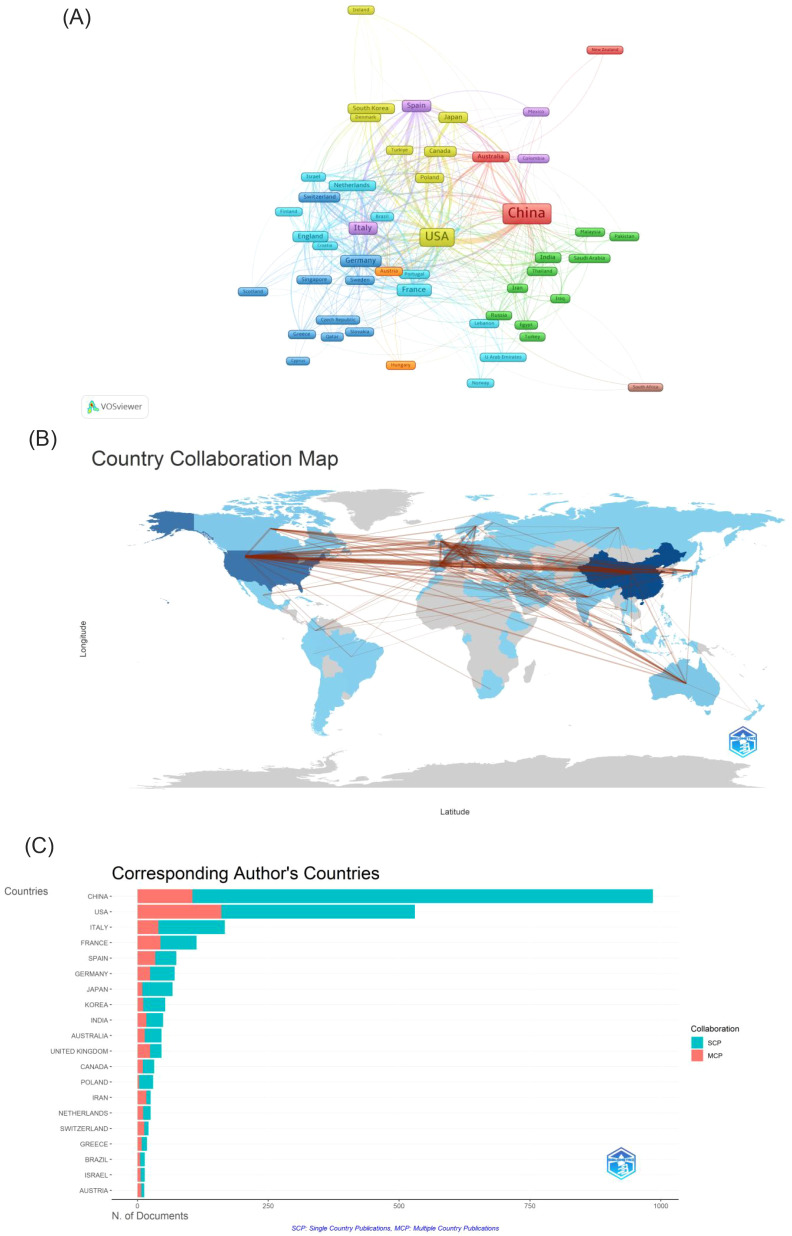
**(A)** Cooperation network of countries in the field. **(B)** A visual map for country collaboration. **(C)** Number of publications from the corresponding author’s country. SCP, Single Country Publications; MCP, Multiple Country Publications.

A total of 88 countries are actively involved in research on immunotherapy resistance in lung cancer, as depicted in **Figure 3**. In 3A, the size of each country’s box represents the total number of publications originating from that nation, while the connecting lines illustrate the strength of collaborative relationships. The analysis highlights a leading collaboration between China and the USA, followed by noteworthy partnerships between the USA and both Italy and France. Other countries tend to have more dispersed and less concentrated collaboration networks. **Table 1** ranks the top ten countries based on publication output. In terms of publication volume, China ranks first with approximately 40.88%, while the USA comes in second with 28.28%.Despite China’s higher number of publications, the USA has accumulated more than double the total number of citations (62,216 compared to 30,997), indicating higher scientific impact. Additionally, both the USA and France have average citation counts exceeding 70 per publication, reflecting the high quality and recognition of research produced in these countries within the field of immunoresistance in lung cancer. The United States, which engages in more international collaboration, demonstrates significantly higher total and average citation rates. This correlation suggests that international collaboration may be more effective in enhancing research impact and recognition than merely increasing publication volume.

**Table 1:** Top 10 most productive countries.

Rank	Country	Publications	Total link strength	Citations	Average citation/publication
1	China	1035	295	30997	29.9
2	the USA	716	584	62216	86.9
3	Italy	222	233	8343	37.6
4	France	160	240	11813	73.8
5	Germany	135	236	6164	45.7
6	Spain	127	238	7510	59.1
7	England	98	195	4900	50.0
8	Japan	98	94	3740	38.2
9	Australia	76	116	3833	50.4
10	South Korea	72	72	3289	45.7

### 3.3 Analysis of institutions

The top 11 most productive institutions are presented in **Table 2**. All are located in either China (n=7) or the USA (n=4). The University of Texas MD Anderson Cancer Center (USA) ranks first with 88 publications, followed by Sichuan University (China) with 65. Regarding citation impact, US institutions—specifically the Memorial Sloan Kettering Cancer Center and the Dana-Farber Cancer Institute—performed better, collectively amassing over 10,000 citations with an average of more than 200 per article.

**Table 2:** Top 11 most productive institutions.

Rank	Institution	Publications	Total link strength	Citations	Average citation/publication
1	Univ Texas MD Anderson Canc Ctr	88	252	6358	72.3
2	Sichuan Univ	65	75	3146	48.4
3	Dana Farber Canc Inst	49	225	10269	209.6
4	ShangHai Jiao Tong Univ	49	91	1449	29.6
5	TongJi Univ	49	61	2199	44.9
6	HuaZhong Univ Sci & Technol	48	35	1423	29.6
7	Mem Sloan Kettering Canc Ctr	48	243	10182	212.1
8	Harvard Med Sch	46	158	5653	122.9
9	Sun Yat Sen Univ	46	64	1621	35.2
10	Chinese Acad Med Sci & Peking Union Med Coll	42	34	694	16.5
11	ZheJiang Univ	42	51	1071	25.5

**Figure 4:**
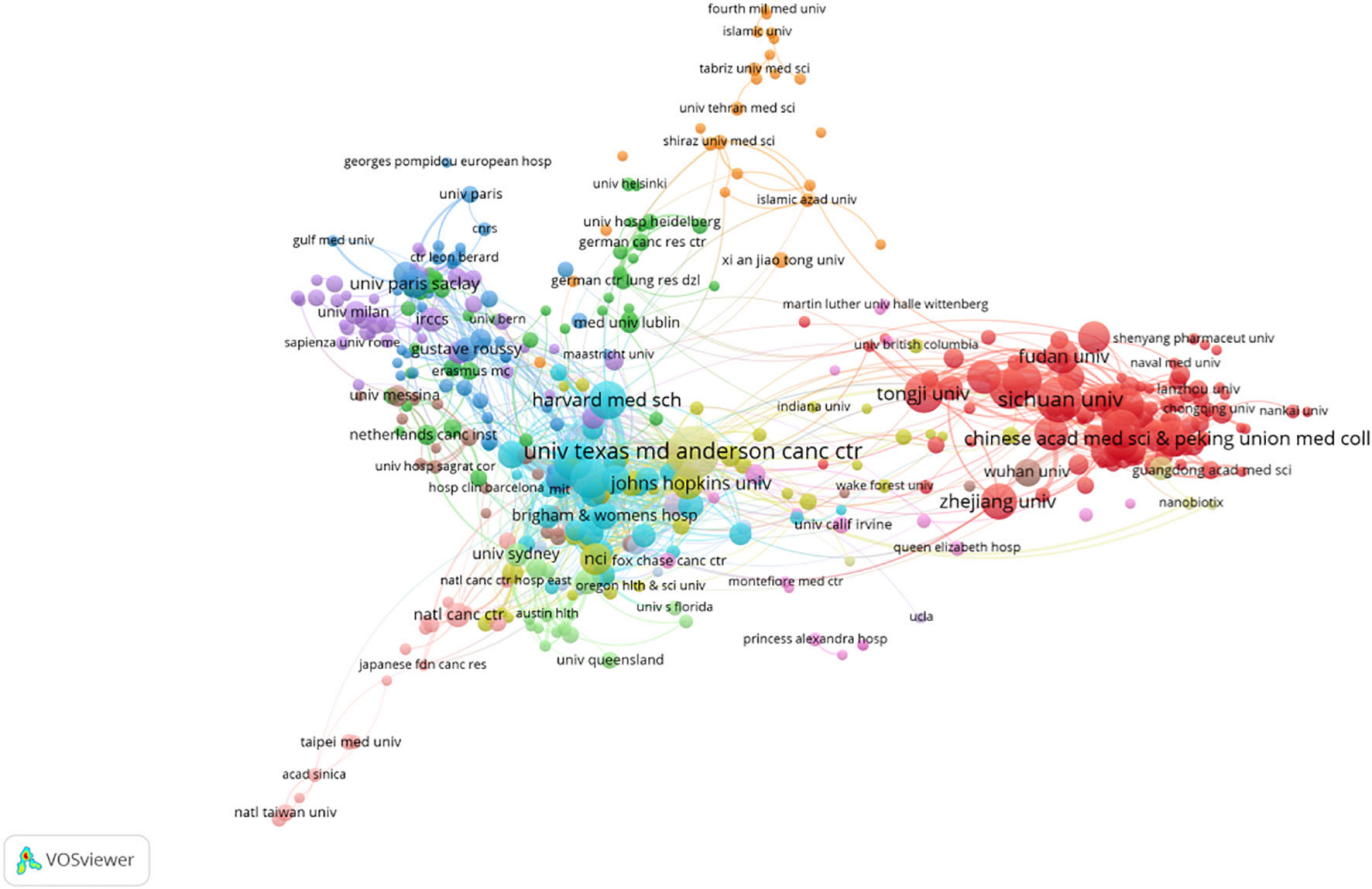
Cooperation network of institutions.

**Figure 4** visualizes the institutional collaboration network, where node size represents publication output and line thickness denotes collaboration strength. Analysis of this network reveals a distinct core-periphery structure. Notably, this pattern indicates a resource agglomeration effect, with a few leading centers acting as major hubs. Conversely, this pattern that collaborative opportunities may be limited for the many institutions on the network periphery. Promoting cross-institutional cooperation is thus a potential strategy for better resource integration and knowledge dissemination.

### 3.4 Analysis of authors

**Figure 5:**
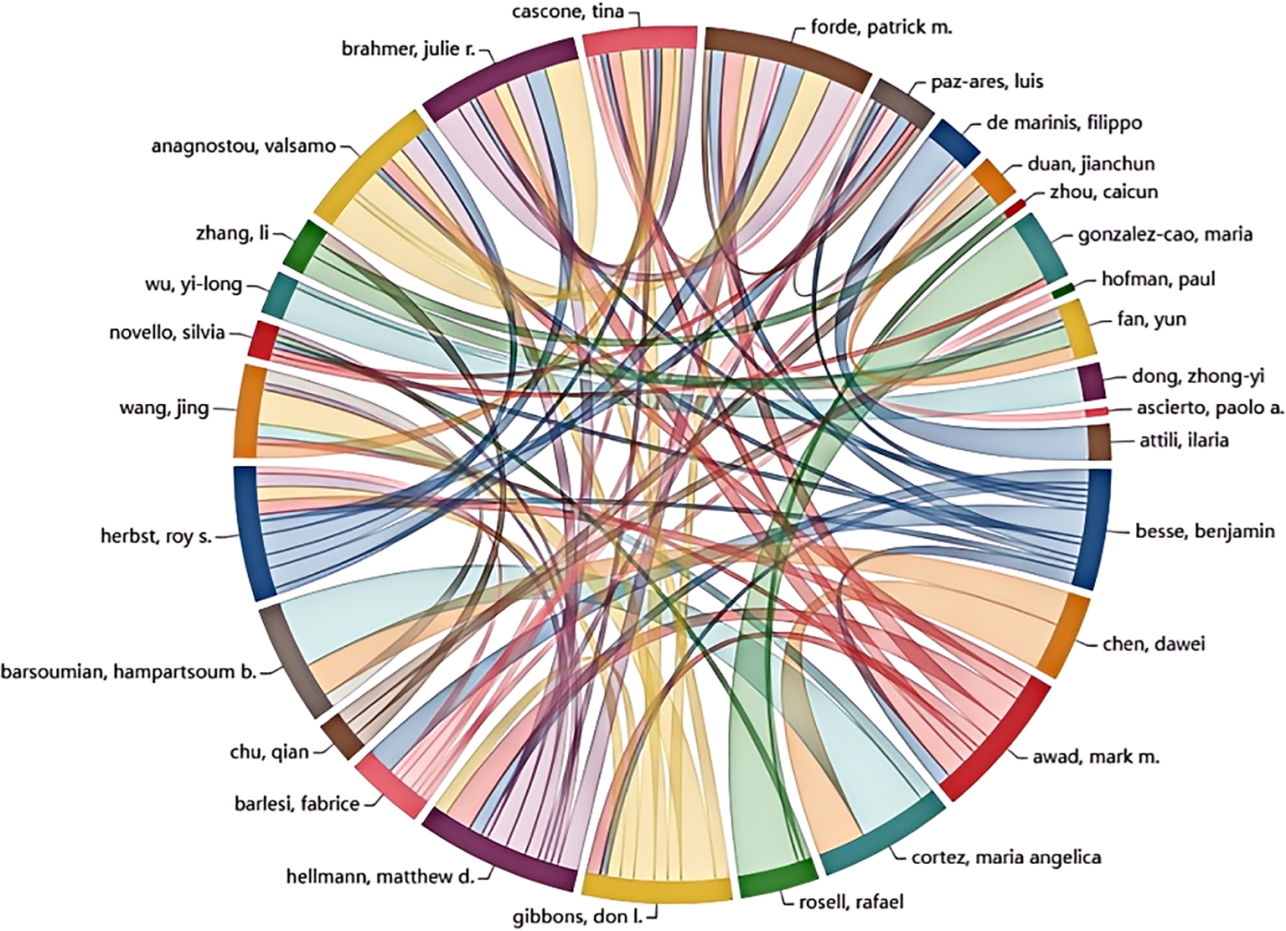
Top 30 authors’ collaboration network map.

A significant number of researchers are actively investigating drug resistance mechanisms in immunotherapy for lung cancer. The current study includes a total of 15,322 researchers, as detailed in **Table 3**, which lists the top ten authors based on publication count and citation impact. To be eligible for this analysis, authors were required to have at least ten publications and 100 citations. Professor Rafael Rosell from the Catalan Institute of Oncology in Spain stands out for his work on KRAS mutation-associated lung cancer and is among the leading contributors in this research area. Scholar Zhou Caicun from Shanghai Lung Hospital in China ranks second in publication volume, with a total of 17 articles. Roy Herbst of Yale Cancer Center in the USA is highly influential, with an impressive total of 5,452 citations, significantly surpassing Don Gibbons, who has 1,324 citations. This highlights Herbst’s impactful contributions to understanding immune resistance in NSCLC. **Figure 5** visually represents the collaboration network among the top 30 authors. It shows that Professor Benjamin Besse from Gustave Roussy Institute in France exhibits substantial collaborative activity with other scholars. However, the overall level of collaboration across the field appears limited, indicating a lack of extensive cooperative efforts among researchers in this domain.

**Table 3:** Top 10 most productive authors.

Rank	Author	Publications	Citations	Average citation/publication	Country
1	Rosell, Rafael	17	1201	70.6	Spain
2	Zhou, Caicun	17	906	53.3	China
3	Herbst, Roy s.	15	5452	363.5	USA
4	Zhang, Li	15	476	31.7	China
5	Gibbons, Don l.	14	1324	94.6	USA
6	Chen, Dawei	13	545	41.9	China
7	Cortez, Maria angelica	12	514	42.8	USA
8	Li, Wei	12	186	15.5	China
9	Salgia, Ravi	12	1234	102.8	USA
10	Wang, Xin	12	489	40.8	China

### 3.5 Analysis of journals

**Figure 6:**
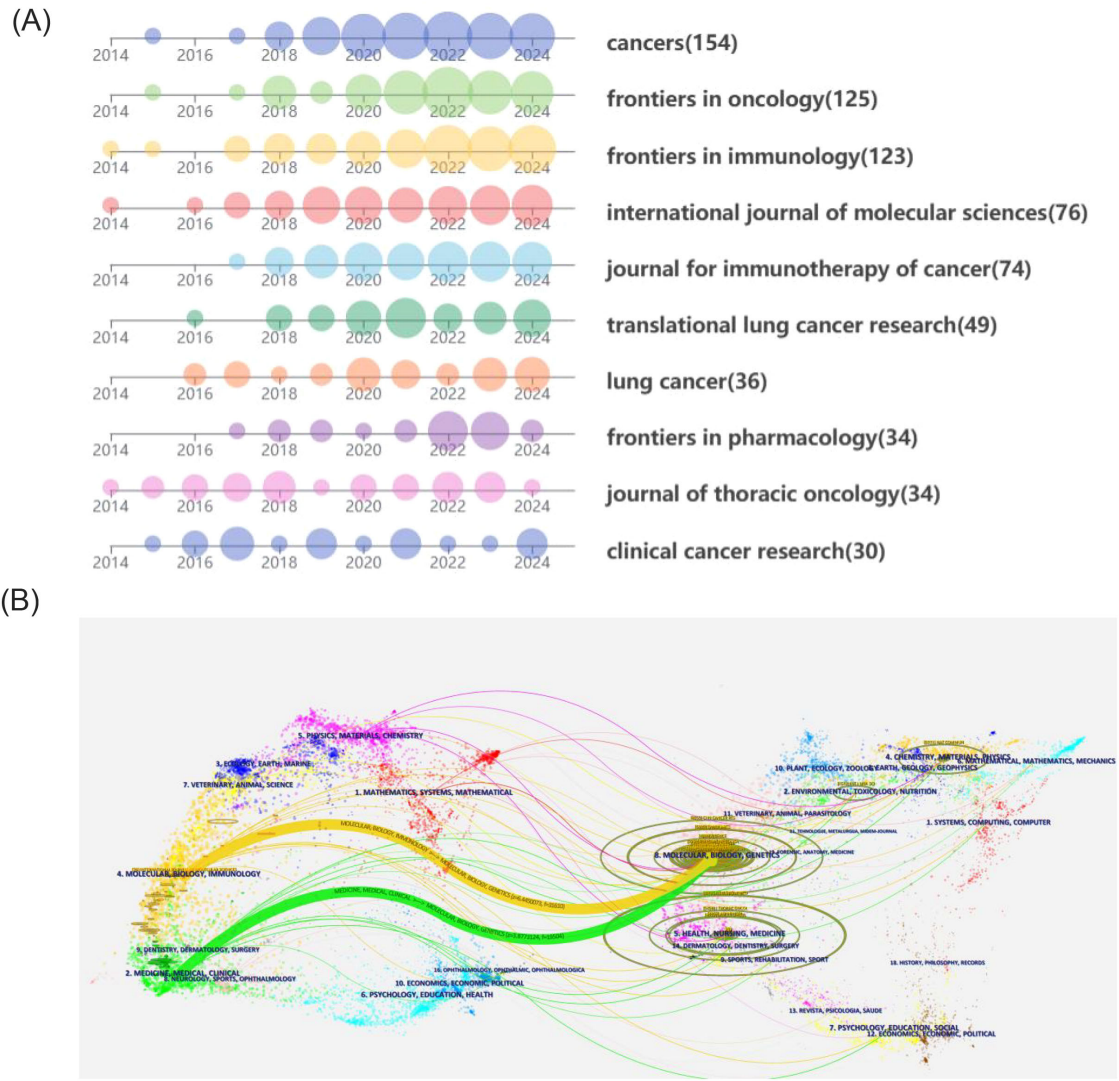
**(A)** Annual publication volume of productive journals. **(B)** Dual-map of journals from publishing fields to cited fields.

**Table 4** lists journals with 30 or more publications relevant to the included literature. Cancers leads with 154 publications, followed by Frontiers in Oncology and Frontiers in Immunology, with 125 and 123 publications, respectively. However, Frontiers in Immunology achieved the highest citation count with 4,583 citations. **Figure 6A** depicts the publication trends in high-volume journals over time. Both the Journal of Thoracic Oncology and Clinical Cancer Research demonstrate a consistent and balanced number of publications per year in this research area.

**Table 4:** Top 10 most productive authors.

Rank	Journal	Publications	Citations	Average citation/publication	JCR/IF
1	Cancers	154	3324	21.6	Q1/4.5
2	Frontiers in Oncology	125	3163	25.3	Q2/3.5
3	Frontiers in Immunology	123	4583	37.3	Q1/5.7
4	International Journal of Molecular Sciences	76	1838	24.2	Q1/4.6
5	Journal for Immunotherapy of Cancer	74	3193	43.1	Q1/10.3
6	Translational Lung Cancer Research	49	695	14.2	Q2/4.0
7	Lung Cancer	36	1043	29.0	Q1/4.5
8	Frontiers in Pharmacology	34	2494	73.4	Q1/4.4
9	Journal of Thoracic Oncology	34	2040	60.0	Q1/21.1
10	Clinical Cancer Research	30	3238	107.9	Q1/10.4

Furthermore, Clinical Cancer Research also boasts the highest average citation count, with an impressive 107.9 citations per article. The majority of the other journals began to publish extensively in this field around 2018. **Figure 6B** maps the disciplinary domains of the citing journals to those of the cited literature. The citing journals are predominantly concentrated in the fields of medicine and clinical research, as well as molecular biology and immunology. On the other hand, the cited journals show a strong focus on molecular biology and genetics. This indicates a connection between fundamental molecular research and its application within clinical and immunological contexts of the study of drug resistance in immunotherapy for lung cancer.

### 3.6 Analysis of citation and co-citation literature

The top 10 cited and co-cited references can be found in **Tables 5**, **6**.The analysis indicates that all highly cited articles are published in journals that are in Q1 and have an IF score of no less than 4, suggesting that the research is of a high caliber within the field. The highest-ranked cited article is a 2017 article in Science by Routy, Bertrand, et al. The researchers found that patients with advanced non-small cell lung and kidney cancers treated with antibiotics experience a disruption of the diversity of the intestinal flora, leading to primary resistance. This may result from the specific microorganisms associated with the absence. The second most prominent review was published in 2018 in Nature by Herbst, Roy S et al. This study offers a thorough and detailed analysis of the factors contributing to the development of the disease, common genetic alterations, and the current status of cancer treatment in NSCLC. The analysis focuses on the fact that Immunotherapy evolved into the standard for advanced treatment, with the promise of applying it to earlier stages in the future. The analysis also notes that it is time to begin to address the issue of inaccurate predictive metrics. The analysis predicts that combination therapies can be used to overcome drug resistance, and it identifies the need to address the inaccuracy of predictors and drug resistance.

**Table 5:** Top 10 most cited papers.

Rank	Title	Citations	Journal	JCR/IF
1	Gut microbiome influences efficacy of PD-1-based immunotherapy against epithelial tumors (10)	3632	Science	Q1/44.8
2	The biology and management of non-small cell lung cancer (11)	3130	Nature	Q1/50.5
3	Comprehensive analyses of tumor immunity: implications for cancer immunotherapy (12)	1631	Genome Biology	Q1/10.1
4	Pan-tumor genomic biomarkers for PD-1 checkpoint blockade-based immunotherapy (13)	1597	Science	Q1/44.8
5	Non-small-cell lung cancers: a heterogeneous set of diseases (14)	1391	Nature Reviews Cancer	Q1/72.5
6	Top 10 Challenges in Cancer Immunotherapy (15)	1310	Immunity	Q1/25.5
7	Myeloid-Derived Suppressor Cells (16)	1295	Cancer Immunology Research	Q1/8.1
8	PD-1 and PD-L1 Checkpoint Signaling Inhibition for Cancer Immunotherapy: Mechanism, Combinations, and Clinical Outcome (17)	1234	Frontiers in Pharmacology	Q1/4.4
9	Adaptive resistance to therapeutic PD-1 blockade is associated with upregulation of alternative immune checkpoints (18)	1185	Nature Communications	Q1/14.7
10	STK11/LKB1 Mutations and PD-1 Inhibitor Resistance in KRAS-Mutant Lung Adenocarcinoma (19)	1106	Cancer Discovery	Q1/30.6

**Table 6:** Top 10 most co-citation papers.

Rank	Title	Citations	Journal	JCR/IF
1	Pembrolizumab versus Chemotherapy for PD-L1-Positive Non-Small-Cell Lung Cancer (20)	417	New England Journal Of Medicine	Q1/96.3
2	Nivolumab versus Docetaxel in Advanced Nonsquamous Non-Small-Cell Lung Cancer (21)	400	New England Journal Of Medicine	Q1/96.3
3	Mutational landscape determines sensitivity to PD-1 blockade in non-small cell lung cancer (22)	351	Science	Q1/44.8
4	Pembrolizumab plus Chemotherapy in Metastatic Non-Small-Cell Lung Cancer (23)	294	New England Journal Of Medicine	Q1/96.3
5	Primary, Adaptive, and Acquired Resistance to Cancer Immunotherapy (24)	289	Cell	Q1/45.6
6	Atezolizumab versus docetaxel in patients with previously treated non-small-cell lung cancer (OAK): a phase 3, open-label, multicenter randomized controlled trial (25)	273	Lancet	Q1/98.4
7	Mutations Associated with Acquired Resistance to PD-1 Blockade in Melanoma (26)	253	New England Journal Of Medicine	Q1/96.3
8	Nivolumab versus Docetaxel in Advanced Squamous-Cell Non-Small-Cell Lung Cancer (27)	250	New England Journal Of Medicine	Q1/96.3
9	Pembrolizumab versus docetaxel for previously treated, PD-L1-positive, advanced non-small-cell lung cancer (KEYNOTE-010): a randomized controlled trial (28)	236	Lancet	Q1/98.4
10	Pembrolizumab for the Treatment of Non-Small-Cell Lung Cancer (29)	233	New England Journal Of Medicine	Q1/96.3

**Figure 7:**
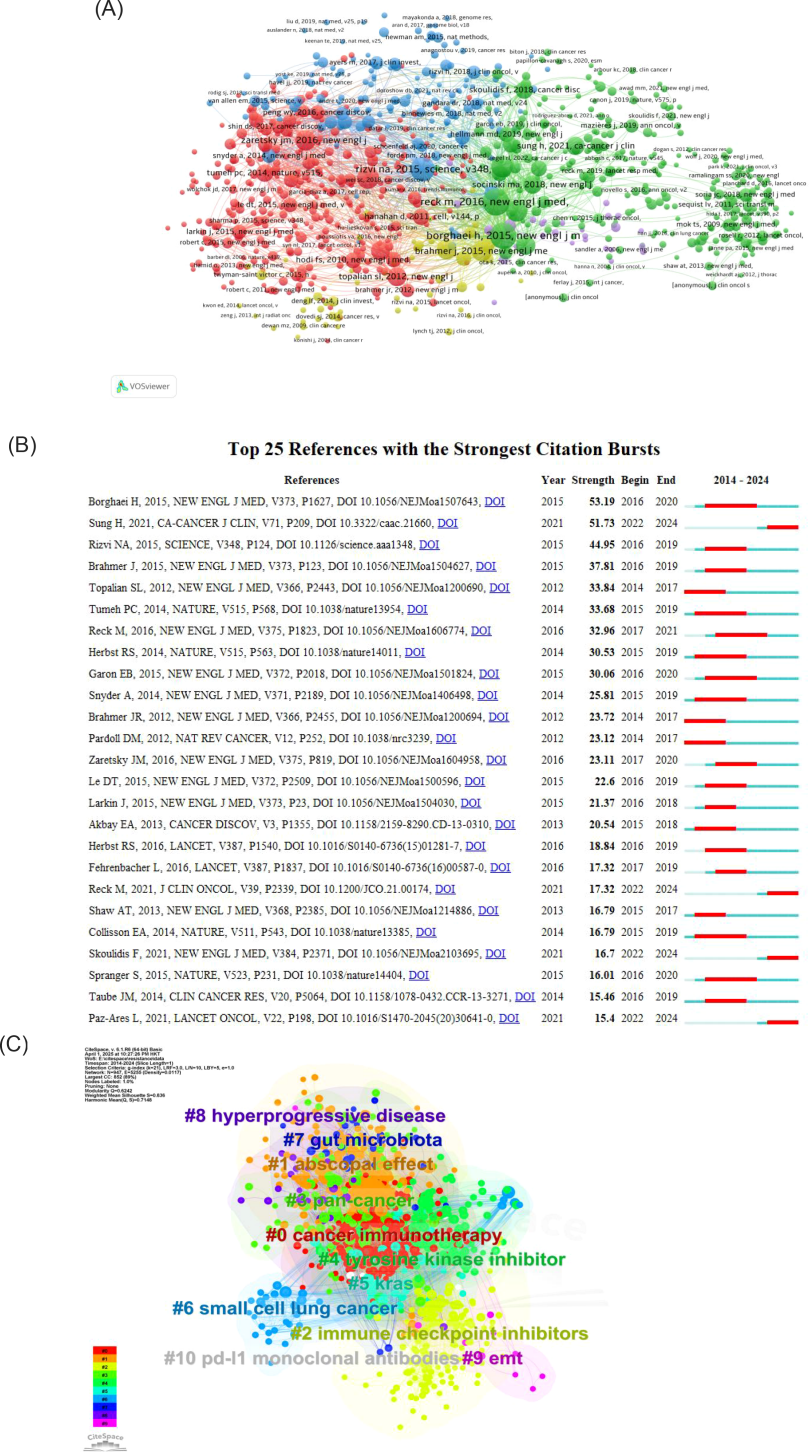
**(A)** Analysis of references. **(B)** Top 25 References with the strongest citation bursts. **(C)** Clustering keywords analysis of references.

The reference visualization map is displayed in **Figure 7**. **Figure 7A** presents a connectivity map generated by VOSviewer, where distinct colors represent disparate clusters of co-cited references, and the size of the circles denotes the number of co-citations. As illustrated in **Figure 7B**, the beginning and end of the citation bursts for co-cited references are displayed, with the red area representing the period of active citation activity and the citation burst strength. The article demonstrating the highest burst strength is a clinical trial by Borghaei, Hossein et al. published their study in the New England Journal of Medicine. This trial compared Nivolumab with paclitaxel in patients with non-squamous NSCLC. It also ranks second in terms of co-citation frequencies, with 400 citations.

**Figure 7C** is a clustering map generated by CiteSpace based on keywords from references. The clusters are primarily divided into ten groups: The following terms are key to understanding the current state of cancer immunotherapy research: #0 cancer immunotherapy, #1 abscopal effect, #2 immune checkpoint inhibitors, #3 pan-cancer, #4 tyrosine kinase inhibitors, #5 KRAS, #6 small cell lung cancer, #7 PD-L1 monoclonal antibodies, #8 gut microbiota, #9 hyperprogressive disease, #10 EMT, and others. The cluster number (#0, #1, etc.) indicates the size of the cluster, with lower numbers corresponding to higher quantities. In general, immune checkpoint inhibitors represent an early research focus in this field. In contrast, the abscopal effect is a more recent area of investigation.

### 3.7 Analysis of keywords

**Figure 8:**
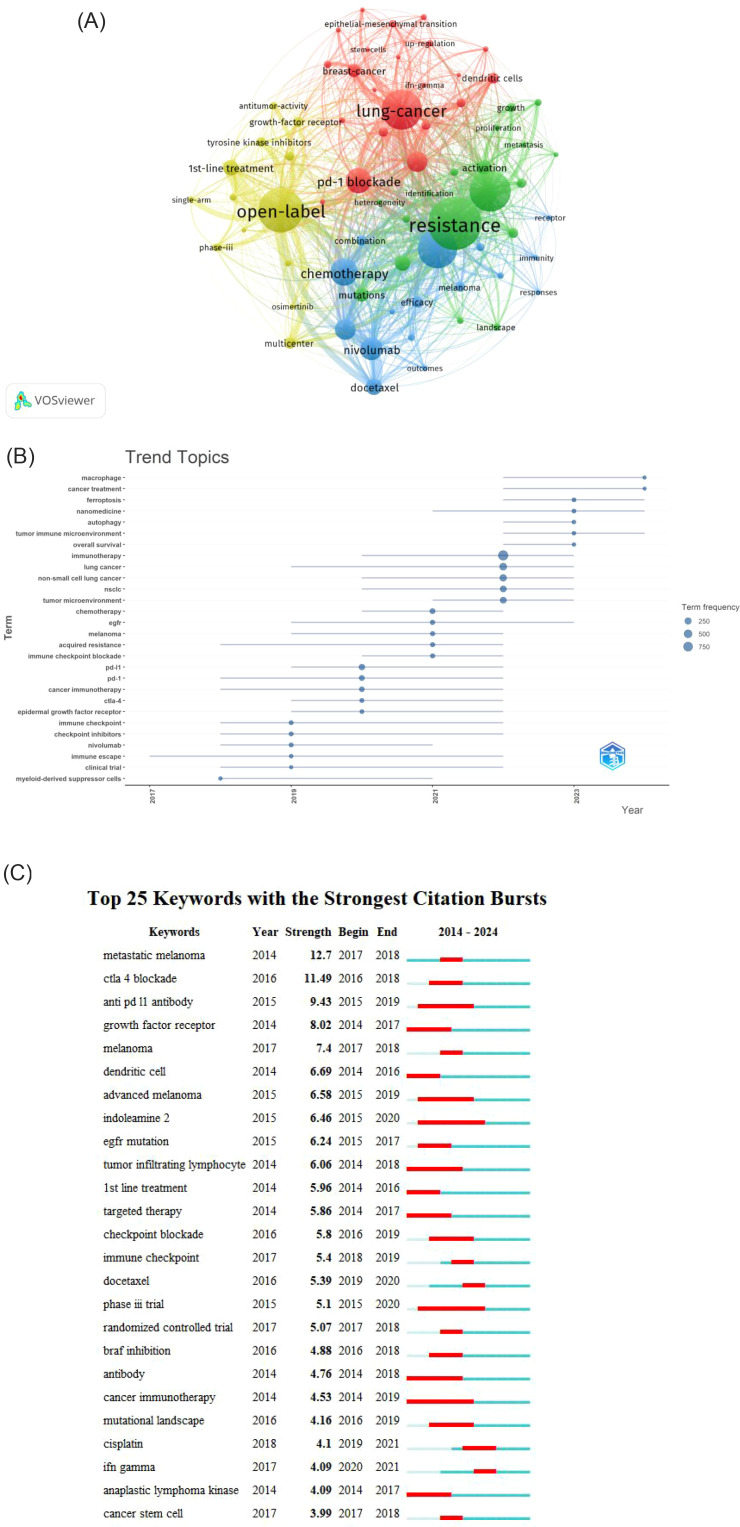
**(A)** Network map of keywords. **(B)** Top 25 Keywords with the strongest citation bursts.

Using VOSviewer for co-occurrence analysis of keywords revealed that, from a total of 7,552 keywords, 860 appeared at least five times, forming the network depicted in **Figure 8A**. Each node represents a keyword, the node size is proportional to the frequency of the keyword’s occurrence. The distance between two nodes approximately indicates their relatedness. The connecting lines represent co-occurrence, with thicker lines indicating stronger association. Nodes are colored by cluster, representing thematically related groups of keywords. In the visualization, The dimensions of each node are proportional to keyword’s occurrence frequency, the thickness of connecting lines reflects the degree of association between the keywords, and node colors represent belonging to different clusters. **Table 7B** lists the top 10 most frequent keywords. After excluding keywords related to the search strategy itself, the most frequent keywords are: open-label (n=469),pd-1 blockade(n=450) chemotherapy (n=352), and T-cells (n=323).These highly frequent keywords highlight the key ongoing research directions within the field of lung cancer immunotherapy resistance.

**Table 7:** (A) Table of Merger strategies for Synonyms and repeated words. (B) Top 10 keywords.

A							
Standard words	Repeat words and synonyms						
lung cancer	lung-cancer, cell lung-cancer, NSCLC,						
resistance	acquired-resistance, drug-resistance						
tumor microenvironment	microenvironment, tumor micro-environment						
pd-l1 expression	expression,pd-l1, ligand 1 expression						
radiation therapy	radiation-therapy, radiotherapy						
pd-1 blockade	blockade, immune checkpoint blockade						
tumor infiltrating lymphocytes	tumor-infiltrating lymphocytes, cd8(+) t-cells						
B							
Rank	keywords	Counts	Total link strength	Rank	keywords	Counts	Total link strength
1	immunotherapy	1010	8381	6	pd-1 blockade	450	4125
2	Lung cancer	943	7870	7	chemotherapy	352	3403
3	resistance	929	6930	8	t cells	363	2797
4	pd-l1 expression	577	4680	9	1st-line treatment	244	2450
5	open-label	469	4243	10	survival	153	1395

The R package “bibliometrix” was used to analyze the temporal evolution of keywords, with the criteria that each year had no fewer than five identified keywords and each keyword was documented a minimum of 15 times. The results are shown in **Figure 8B**. A review of the literature indicates a shift in topical hotspots. From 2014 to 2019, the focus was on clinical trials and specific medications like nivolumab, as well as the immune checkpoint. Between 2019 and 2021, there was a gradual evolution towards immunotherapy targets such as CTLA-4, PD-1, and PD-L1. From 2021-2023, the most prevalent keywords show a trend towards a comprehensive analysis of chemotherapy in combination with immunotherapy, particularly focusing on the emergence of resistance to chemotherapy and the role of immune checkpoint inhibitors in lung cancer prognosis. In 2024, the prominent keywords are “tumor immune microenvironment,” “autophagy,” and “ferroptosis,” highlighting current research trends aimed at elucidating the molecular immune mechanisms underlying immunotherapy and the mechanisms by which immunotherapy leads to the demise of tumor cells.

**Figure 8C** presents a keyword with the strongest citation bursts by Citespace, illustrating keywords with significant prominence from 2014 to 2024. The overall intensity of keywords experiencing high outbreaks exceeds a value of 3. However, all notable outbreak keywords are cut off before 2021. Notably, after the keyword “chemotherapy,” the prominent outbreak keywords shift to “tumor microenvironment,” indicating a transition in research focus from clinical studies to investigations of the molecular mechanisms underlying the tumor microenvironment. As shown in **Figure 8B**, “tumor microenvironment” (TME) emerges as a key focus following chemotherapy, reflecting a shift in research priorities from clinical applications towards understanding the molecular and mechanistic aspects of tumor biology.

## 4 Discussion

### 4.1 General information

Over the past decade, the annual number of publications in this field has increased dramatically, from 21 to 476. The past seven years have witnessed particularly rapid growth, with the annual publication count exceeding 100 and citations surpassing 10,000, accounting for 95% of all publications within that period. Globally, 88 countries have shown active involvement in this area. China, the USA, Italy, and France have made substantial contributions, with China and the USA exhibiting the strongest collaborative ties. The University of Texas MD Anderson Cancer Center in the USA leads in article publication count, while the Dana Farber Cancer Institute, also in the US, excels in overall citation count. While China leads in both total publications and citations, the USA demonstrates superior citation metrics, particularly in total citations and average citations per document. Considering the highly cited articles, US research focuses on two main areas: clinical trials of immunotherapy drugs (13) and targeted biomarkers of immunotherapy resistance (18). Conversely, Chinese research centers on the molecular mechanisms of drug resistance in immunotherapy, including alterations in the immune microenvironment and specific cell death pathways induced by immunotherapy (30, 31). Despite these contributions, international collaboration remains limited. Moving forward, it’s crucial for countries to strengthen cross-regional institutional cooperation, integrate resources, and leverage their individual strengths to further advance the field.

This study reveals a markedly uneven global research landscape in this field, characterized by the dominance of China and the United States. This pattern reflects fundamental differences in the two countries’ scientific development pathways and resource allocation strategies. China’s high research output can be largely attributed to its national-level strategic prioritization, a substantial researcher workforce, and extensive clinical resources—a combination that exemplifies a “scale-driven” model. However, its research influence remains comparatively limited, indicating a need to further encourage original investigations and in-depth mechanistic studies. In contrast, the United States has established a leading position in research impact, underpinned by its capacity to attract global scientific talent, a strong foundation in basic research, and a dynamic ecosystem for translating research into application—features that align with an “impact-driven” model. This divergence underscores the importance of fostering more substantive international collaboration in the future. Integrating China’s scale advantages with the U.S. capacity for innovation could help catalyze breakthrough advances in the field.

In the landscape of scientific journals, the majority of publications are currently focused on clinical, immunological, and medical areas, representing macro-level research. Looking ahead, it is anticipated that these journals will increasingly publish studies delving into molecular biology and other fundamental medical disciplines. This shift seeks a deeper mechanistic understanding of immune drug resistance, thereby advancing the foundational knowledge necessary for developing more effective therapeutic strategies.

The three most prolific researchers in the field are Rafael Rosell (Spain), Caicun Zhou (China), and Roy S. Herbst (the USA).Rosell’s work centers on the diagnosis and treatment of NSCLC, encompassing immunotherapy combinations with oncolytic viruses and gene mutation screening in NSCLC patients (32–34). Zhou’s research focuses on immune escape mechanisms. He has explored the combination of radiotherapy and anti-PD-L1 antibody therapy in NSCLC to effectively reduce drug resistance (35). Additionally, he investigates recurrence risk assessment in SCLC, identifying Galectin-9 (Gal-9) in TME and (tumor-infiltrating lymphocytes)TILs as a promising predictive immune biomarker, with Gal-9 expression levels correlating significantly with the immune risk score (36). Herbst’s research has focused on the role of neutrophils in immunotherapy resistance in NSCLC (37). He has also investigated immunosuppressive receptors like PD-1, LAG-3, and TIM-3 in the context of immunotherapy, finding them associated with a pro-apoptotic T cell phenotype and that elevated LAG-3 expression doesn’t correlate with PD-1 axis blockade (38).While some author collaboration exists, overall it is limited. A broad and extensive collaborative network within this field is currently lacking.

The analysis of cited and co-cited literature demonstrates the background and development of research in the field. The integration of highly cited literature and high-frequency keywords offers a comprehensive overview of the state-of-the-art research in the field. The focal points of this field have undergone a gradual transition from early clinical trials and specific medications to immunotherapy targets, chemotherapy combination immune effects and resistance issues, and then to the current a thorough examination of the molecular mechanisms of immunotherapy and the way of tumor cell death in TME. The importance of the TME has prompted the realization that comprehensive studies are necessary, encompassing not only the tumor cells themselves but also the intricate interplay among the extracellular matrix, signaling molecules, immune cells, blood vessels, and lymphatic vessels within the TME. Consequently, research at the fundamental level has become a highly sought-after topic in contemporary discourse.

### 4.2 Mechanisms

Our bibliometric analysis provides a data-driven roadmap to the most salient mechanisms of immunotherapy resistance. The keyword evolution map **Figure 8** clearly demonstrates a temporal shift from broad clinical terms to specific molecular concepts, with tumor microenvironment, autophagy, and ferroptosis emerging as the most recent and prominent hotspots. Furthermore, co-citation cluster analysis **Figure 7C** underscores the centrality of immune checkpoint inhibitors, the gut microbiome (#8),and hyperprogressive disease (#9) within the intellectual structure of the field. In this section, we synthesize these bibliometric signals with the foundational literature to discuss the key resistance mechanisms that are currently shaping the research landscape.

Mechanisms of immunotherapy resistance in lung cancer primarily involve two key aspects: TME and internal tumor factors.

TME is considered a key factor in immunotherapy resistance (39) and contains immune cells that demonstrate both anti-tumor and pro-tumor effects. Intratumoral immune effector cells (such as CD4+ and CD8+ T cells) create an anti-tumor inflammatory microenvironment that inhibits tumor growth early in tumor progression (40). However, persistent stimulation of tumor antigens resulted in impaired infiltration, dysfunction, exhaustion, and reduced memory cell formation in these effector cells. This, in turn, promotes tumorigenesis, establishes an immunosuppressive microenvironment, and ultimately initiates the process of drug resistance (41). In immunosuppressive cells, Tregs regulate the activation and proliferation of cytotoxic CD8+ T cells and effector CD4+ T cells (42). Bone marrow-derived myeloid-derived suppressor cells (MDSCs) inhibit immune responses and impair the function of T cells and natural killer (NK) cells (43).Tumor-associated macrophages reduce T cells antigen presentation and release immunosuppressive factors (44).Together, these processes promote tumor growth, metastasis, and immune evasion, leading to drug resistance responses. In addition to immune cells, cancer-associated fibroblasts (CAFs) also play a pivotal part in the TME.CAF remodels extracellular matrix by secreting cytokines such as transforming growth factor beta (TGF-β) and vascular endothelial growth factor (VEGF). These cytokines not only provide material support to tumor cells, but also induce immune cell dysfunction, promote tumor angiogenesis, induce tumor cell escape to evade immune surveillance, and ultimately exacerbate immune resistance (45).

Intratumor factors have been shown to have a central role in mediating resistance to immunotherapy in lung cancer, primarily through gene mutations, impaired antigen presentation, and epigenetic modifications. NSCLC often harbors mutations in driver genes such as EGFR, ALK, and KRAS. These genetic alterations can facilitate immune evasion via distinct mechanisms. For example, an EGFR-sensitive mutation (exon 19 deletion) with low PD-L1 expression (6%-10%) may suppress the interferon-gamma (IFN-γ) pathway through EGFR signaling, reducing immune activation. Additionally such mutations can promote an immunosuppressive microenvironment through the secretion of cytokines such as TGF-β and IL-10, which attract Tregs and MDSCs, thereby inhibiting effector T cell responses (46). Additionally, STK11/LKB1 mutations cause resistance to PD-1 blockade in KRAS-mutated lung adenocarcinoma (19).The reduced antigen-presenting ability of tumor cells is characterized by abnormalities in MHC molecules. Abnormalities in MHC molecules, particularly a reduction or loss of MHC class I expression, prevent recognition by CD8+ T, leading to resistance to immunotherapies (47). Epigenetic modifications further contribute to immune resistance by regulating gene expression through mechanisms like DNA methylation, non-coding RNA expression, and post-transcriptional changes. These alterations promote tumor invasiveness and enable tumor cells to evade immune surveillance, thereby diminishing the efficacy of immunotherapy (48, 49).

Individual patient factors, such as sex, age, and smoking history, contribute to variability in responses to immunotherapy. Age-related changes impact the immune system. For instance, in elderly individuals, CD4+ T cell responses tend to favor the production of inflammatory effector T cells prone to damage. This shift hinders the evolution of long-lived memory cells, ultimately weakening the overall immune response (50). Furthermore, alterations in the gut microbiome, potentially resulting from antibiotic and hormonal drug use, can increase susceptibility to immune resistance (8).The presence of comorbidities, such as diabetes, has been demonstrated to impede the efficacy of immunotherapy and diminish the patient’s capacity to derive benefit from treatment (51).

A thorough understanding of the mechanisms driving these factors, combined with the development of targeted therapies, offers significant potential to improve the effectiveness of immunotherapy. This focus will be the direction of our future research efforts.

### 4.3 Biomarkers for predicting the efficacy of lung cancer immunotherapy

Our analysis of the most cited and co-cited literature **Table 5** and **Table 6** reveals that the search for predictive biomarkers constitutes a core and highly influential research theme. Landmark papers defining PD-L1 expression (20, 28), tumor mutational burden (TMB) (13, 22), and the influence of the gut microbiome (10) rank among the most frequently cited works. This underscores the field’s intense effort to identify patients who will benefit from immunotherapy.

International Association for the Study of Lung Cancer Pathology has identified several biomarkers that can predict patient responses to immunotherapy in lung cancer (52).These include PD-L1 expression levels (53), tumor mutation burden(TMB) (54),TIL (55),interferon levels(IFN) (56), and the neutrophil to lymphocyte ratio (NLR) (57). Additionally, molecular features such as gene mutations and signaling pathway alterations provide further insights. For example, reduced tumor antigen presentation associated with certain human leukocyte antigen class I (HLA-1) variants correlates with resistance to immunotherapy (58). Patients with ALK rearrangements and EGFR mutations tend to respond poorly to ICIs (59), and triggering of WNT/β-catenin pathway has been associated with immunotherapy resistance (60). However, a significant challenge remains in the lack of standardized assessment criteria for these biomarkers, leading to conflicting findings—such as the inconsistent results regarding blood tumor mutational load (bTMB) and its impact on patient survival and treatment efficacy (52).To fully realize their clinical potential, further research is necessary to establish standardized evaluation methods. In-depth investigation into the mechanisms underlying these biomarkers, coupled with the development of targeted therapeutic strategies, holds substantial promise for enhancing immunotherapy effectiveness. This direction represents a key focus of our future research efforts.

### 4.4 Methods for improving the efficacy of lung cancer immunotherapy

As discussed previously, numerous mechanisms can limit the effectiveness of immunotherapy. Current research is actively focused on overcoming these limitations, and the following sections will provide an overview of recent findings and advancements in this field.

Combination therapies primarily involve integrating immunotherapy with chemotherapy and radiotherapy. The rationale for combining immunotherapy arises from the intricate and heterogeneous nature of TME, the varied immune escape mechanisms utilized by tumor cells, and the restricted effectiveness of immunotherapy when administered as a standalone approach. The combining different anti-tumor approaches can synergistically enhance anti-tumor immune responses, thereby expanding the scope of tumor control (61). A substantial body of research has demonstrated that patients receiving combined immunotherapy and chemotherapy have exhibited significantly prolonged survival times in comparison to those treated with chemotherapy alone (8, 62, 63).The combination of chemotherapy and immunotherapy exhibits a multifaceted synergistic effect, operating through several mechanisms. These include the direct elimination in tumor cells, the augmentation in T cell proliferation and functionality, the mitigation in immunosuppressive substances secreted by tumors, the facilitation of antigen presentation, and the amplification in comprehensive tumor suppression response (64). It is crucial to carefully optimize the dosage and sequence of chemotherapy and immunotherapy to effectively counteract resistance and maximize therapeutic efficacy. Furthermore, radiotherapy plays a pivotal role in modulating the TME. It has been demonstrated to promote the processing and presentation of tumor antigens, enhance the function in dendritic cells, and promote anti-tumor immune responses. Furthermore, radiotherapy has been shown to enhance the infiltration of CD8+ T cells, which are pivotal effector cells in the anti-tumor immune response. This increased infiltration can, in turn, enhance the efficacy of immune checkpoint inhibitors. The combination of immune therapy and radiotherapy has been demonstrated to yield substantial improvements in survival outcomes for patients with lung cancer (65–67).

The most prevalent dual immune combination strategy involves simultaneous blockade of multiple immune checkpoints, such as combining PD-1/PD-L1 inhibitors with CTLA-4 inhibitors. In this approach, CTLA-4 inhibitors primarily promote T-cell activation during the initial priming phase, while PD-1/PD-L1 inhibitors are involved in reactivating exhausted effector T cells at later stages. This dual blockade is crucial for preventing cancer cells from evading immune destruction by overcoming immune suppression mechanisms. By targeting these two checkpoints simultaneously, this strategy also modulates signals to antigen-presenting cells and diminishes the immunosuppressive effects of Tregs and MDSCs (68).This approach increases tumor sensitivity to PD-L1 blockade and helps overcome immune resistance (69). This strategy has shown significant efficacy in first-line treatment studies (70).

Oncolytic viruses (OVs) remodel TME by orchestrating a series of crucial changes that tip the balance towards anti-tumor immunity. This includes reducing the presence and activity of Tregs and MDSCs, inhibiting the production of immunosuppressive factors like TGF-β, and simultaneously boosting the release of immunostimulatory cytokines. These actions culminate in the creation of a more favorable microenvironment that supports effective immune cell infiltration and function, ultimately helping to overcome existing immune resistance mechanisms (71).

Nanomedicine has demonstrated significant potential in modulating the TME to enhance antigen presentation and stimulate immune activation. For example, SGT-53, a nanomedicine delivering a plasmid encoding human wild-type p53, has been shown to restore effective immune responses against lung cancer cells. This restoration occurs through the reduction of immunosuppressive cell populations within the TME and the downregulation of immunosuppressive molecules, which helps mitigate immune resistance. Consequently, these effects promote an increase in CTL activity, thereby strengthening the anti-tumor immune response (72).

Tumor vaccines are a novel form of immunotherapy designed to elicit a spontaneous anti-tumor immune response by presenting various tumor-related antigens, such as tumor cells, tumor-associated proteins, and exosomal components, to the immune system (73). Ideal tumor vaccines utilize personalized tumor antigens, which are less likely to induce immune tolerance, thereby enhancing their efficacy. However, the widespread application of such personalized vaccines faces significant challenges, including the lengthy production process, high costs associated with vaccine design, and the expenses involved in generating individualized neoantigen libraries and assays. These factors currently represent major obstacles to the broader utilization of tumor vaccines in clinical practice (74).

Adoptive Cell Therapy(ACT), a cellular immunotherapy in which special immune cells are modified and expanded and fed into the patient’s organism to stimulate his or her immune system to kill tumor cells, has been a significant advantage in the treatment of hematological tumors (75). TIL-based adoptive cell therapy can benefit patients with advanced NSCLC that is resistant to PD-1 inhibitors. This approach has demonstrated a favorable safety profile and represents a novel therapeutic strategy for metastatic lung cancer (76).

### 4.5 Limitations

This study has several limitations. First, by including only English publications, we may have under-represented research from non-English speaking regions, potentially amplifying the apparent dominance of China and the USA. Second, citation-based metrics inherently favor older publications due to the time required to accumulate citations; thus, recent high-impact work from 2023–2024 may be undervalued. Third, with a data cutoff at the end of 2024, our analysis cannot capture the most recent developments in this rapidly evolving field. Notwithstanding these limitations, this study provides valuable insights into the characteristics and trends in the field of drug resistance to immunotherapy in lung cancer.

### 4.6 Research prospects and implications for clinical translation

This bibliometric analysis not only maps past achievements but, more importantly, illuminates a path for future progress. By analyzing keyword evolution, co-citation patterns, and high-impact studies, we have identified critical research gaps and derived future directions with direct relevance to clinical practice.

First, from correlation to causation: addressing clinical heterogeneity in the TME. Although the TME is a central research focus, current studies remain largely descriptive. The profound heterogeneity of the TME is a key driver of variable responses to immunotherapy, representing a major clinical challenge. Future work must move beyond associative observations to elucidate the causal mechanisms through which specific TME components drive resistance. This will require integrating longitudinal clinical samples with multi-omics technologies to identify actionable therapeutic targets. The clinical implication is clear: successfully deconvoluting TME subtypes will enable the matching of optimal combination therapies such as agents targeting immunosuppressive cancer-associated fibroblasts or specific macrophage subsets to individual patients, advancing the goal of precision immunotherapy.

Second, from monotherapy to rational combinations: next-generation strategies to overcome resistance. Keyword evolution reflects a field shifting from single-agent immune checkpoint inhibitors toward combination therapy. However, current strategies remain relatively narrow in scope. Co-citation clustering highlights emerging immune checkpoints (e.g., LAG-3, TIM-3), TME metabolic pathways (e.g., adenosine), and non-apoptotic cell death mechanisms (e.g., ferroptosis) as promising frontiers. The clinical significance of these findings is their potential to provide new therapeutic blueprints for patients resistant to existing immunotherapies. Translating these strategies into clinical validation is essential for overcoming current efficacy plateaus.

Finally, from generic to predictive: ushering in a new era of biomarker development. While biomarkers such as PD-L1 and TMB represent key research themes, their predictive performance remains suboptimal, revealing a critical gap. The future lies in developing dynamic, integrated multi-dimensional biomarker systems. This entails using liquid biopsy for the non-invasive monitoring of resistant clone evolution and building predictive models that incorporate genomic, transcriptomic, and microbiome data. The potential clinical impact is substantial: such tools would enable more precise patient stratification, minimize toxicity and costs from ineffective treatments, and ultimately maximize the benefit of immunotherapy.

In summary, this study underscores that the bridge between research output and clinical impact is built through translational science. As illustrated in **Table 5**, landmark, highly-cited studies consistently originate from a deep understanding of clinical problems (such as resistance) and are solved through close collaboration between basic and clinical research. Therefore, enhancing the clinical value of future work depends not merely on increasing output volume, but on strengthening the translational research cycle—from bedside to bench and back—ensuring that every study is designed to address a defined clinical need.

## 5 Conclusion

This study comprehensively and systematically reviews research trends in lung cancer immunotherapy resistance over the past ten years. The findings provide valuable insights into global research developments, elucidating the current landscape of immunotherapy resistance in lung cancer. By summarizing key trends and mechanisms, this study establishes a foundation for future research, aiding scholars in identifying innovative directions and effectively navigating the field. Lung cancer immune resistance is characterized by a complex and heterogeneous array of mechanisms, and optimizing strategies to target various resistance pathways could significantly enhance patient prognoses. Addressing these intricate systems necessitates ongoing academic efforts, including in-depth exploration of underlying mechanisms, integration of emerging technologies, and enhanced international collaboration. Through interdisciplinary innovation and clinical translation, it is expected that future advancements could overcome drug resistance challenges, ultimately improving survival outcomes for patients.

## Data Availability

The original contributions presented in the study are included in the article/supplementary material. Further inquiries can be directed to the corresponding authors.
